# Effect of Silibinin on Maspin and ERα Gene Expression in MCF-7 Human Breast Cancer Cell Line

**Published:** 2017-04-01

**Authors:** Maryam Karimi, Hossein Babaahmadi-Rezaei, Ghorban Mohammadzadeh, Mohammad-Ali ghaffari

**Affiliations:** 1 *Dept. of Clinical Biochemistry, Faculty of Medicine, Ahvaz Jundishapur University of Medical Sciences, Ahvaz, Iran*; 2 *Hyperlipidemia Research Center, Dept. of Clinical Biochemistry, Faculty of Medicine, Ahvaz Jundishapur University of Medical Sciences, Ahvaz, Iran*; 3 *Cellular and Molecular Research Center, Dept. of Clinical Biochemistry, Faculty of Medicine, Ahvaz Jundishapur University of Medical Sciences, Ahvaz, Iran*

**Keywords:** Breast cancer, ERα, Maspin, MCF-7 cell line

## Abstract

**Background and objective:**

According to reports, a serine protease inhibitor (Maspin) suppresses metastasis, invasion and angiogenesis in breast and prostate cancers. Silibinin is a natural polyphenolic flavonoid with anti-cancer activity. We assessed the effects of silibinin on cell viability, maspin and ERα gene expression in MCF-7 cell line.

**Methods:**

The human MCF-7 breast cancer cell line was cultured in Dulbecco’s Modified Eagle’s Medium (DMEM) and treated with different concentrations of silibinin (100-600 μg/mL) for 24, 48 and 72 hours. The cytotoxic effect of silibinin on MCF-7 viability was determined using Methyl-Thiazolyl-Tetrazolium (MTT) assay by IC50 determination. The fold changes of Maspin and ERα expression were determined by reverse-transcription real-time Polymerase Chain Reaction (PCR). All experiments on the cells were performed in triplicates.

**Results:**

The maximum inhibitory effect of silibinin on cell viability was observed at 600 μg/mL after 72-hour incubation (p = 0.001). Incubation of the cells with silibinin for 48 and 72 hours significantly decreased IC50 values to 250 and 207 μg/mL (p = 0.005 and p= 0.006), respectively. The expression of maspin and ERα in the treated cells compared to controls was significantly decreased following treatment with different concentrations of silibinin during a 24-hour period.

**Conclusions:**

Silibinin reduces both maspin and ERα gene expression in MCF-7 cell line. The therapeutic effect of silibinin on the treatment of breast cancer may be mediated by the reduction of ERα expression. For verifying this hypothesis and the possible therapeutic implication of silibinin on breast cancer, further studies in this direction are necessary.

## Introduction

Maspin (mammary serpin), a relatively unique member of the serine protease inhibitor family, was originally identified from normal mammary epithelial cells by hybridization assay, according to its expression at the mRNA level (1, 2). Generally, maspin induces apoptosis, and inhibits progression of cancer cell, angiogenesis and invasion, both in cell culture and animal models (3-5). The Maspin gene expression in human breast cancer is paradoxical, since, under-and overexpression have been reported (6, 7). On the other hand, down-regulation of maspin in breast carcinoma is associated with a higher risk of distant metastasis (8-10). Interestingly, expression of maspin has a predictive importance in ER-α positive postmenopausal breast cancer (11). Generally, maspin is highly expressed in myoepithelial cells of normal breast tissue (1). 

The estrogen receptor is a member of the steroid and thyroid nuclear receptor superfamily that mediates several biological functions, which are essential for reproduction, cardiovascular, skeletal, and nervous system function. Currently, studies have shown that there are two distinct subtypes of estrogen receptor, referred as α and β. Most of the growth-promoting effects of estrogens in breast cancer have been related to ERα. Bieche et al. demonstrated a statistical association between decreased maspin expression and positive estrogen receptor. The ERα-negative breast cancers had better expression of maspin than ERα-positive breast tumors (12). Liu et al. reported that tamoxifen induces the expression of maspin through an estrogen receptor in breast cancer (13). This result was confirmed by similar action in prostate cancer cells with estrogen receptor signaling pathway (14).

Chemotherapy for breast cancer usually has a strong cellular cytotoxicity and serious side effects. Furthermore, attention has been paid to the combination of natural phytochemical agents. Phytochemicals from fruits and vegetables, are referred to as chemo-preventive agents, and include genistein, lycopene, capsaicin, curcumin, ellagic acid, ursolic acid, silymarin, catechins, and other compounds. Silibinin, a major bioactive component of silymarin, originally was extracted from milk thistle, is a mixture of polyflavonoids and traditionally used as an anti-hepatotoxic agent. Recently, silibinin has been considered as an obvious anti-cancerogenic agent against different tumor cells, such as hepatocellular carcinoma (15), prostate (16), renal (17), colon (18), lung (19), and skin cancers (20). Currently, clinical trial studies of silibinin in humans are underway for treatment of prostate cancer, and a completed phase I and II study has shown no toxic effects (21). While molecular mechanisms for chemo-preventive effects of silibinin have not been completely understood, the mechanism of its anti-proliferative effects on breast carcinoma was reported (22). Regarding the key role of maspin and ERα in the biology of breast cancer, in this study, we aimed at determining whether silibinin affect maspin and ER-α gene expression in MCF-7 human breast cancer cell line.

## Materials and Methods

**Chemicals and Reagents**

Silibinin, with a purity of 98%, was purchased from Sigma-Aldrich, USA. Dulbecco's modified Eagle's medium (DMEM) (Bioidea, Iran), 3-(4,5-Dimethylthiazol- 2-yl)-2,5-diphenyltetrazolium bromide (MTT) (Johnson Motthey, England), fetal bovine serum (Gibco, USA), MCF-7 cell line (Pasteur Institute of Iran), penicillin (Bioidea, Iran), streptomycin (Bioidea, Iran), First-Strand C.DNA Synthesis kit (Takara, Japan), SYBR Green PCR Master Mix (Takara, Japan) and RNeasy® Plus Mini Kit (Quiagen, Maryland, US) were obtained from Iran, Tehran. A stock solution of silibinin with concentration of 100 mM in dimethyl sulfoxide was made and kept at −20˚C. 

**Cell Culture and Cytotoxicity**

A human breast cancer cell line (MCF-7) was grown in DMEM, containing 10% FBS and antibiotics (penicillin G, 10000 unit/mL, and streptomycin 10000 µg/mL), and incubated in a humidified atmosphere (37°C, 5% CO_2_, and 95% air). After growing a sufficient amount of cells, the cytotoxic effect of silibinin on the MCF-7 was examined by the MTT assay after 24, 48, and 72 hours of treatment. Briefly, 5×10^3^ cells/well was cultivated in a 96-well culture plate. After a 24-hour incubation at 37°C, cells were treated in triplicates with different concentrations of silibinin (100 to 600 μg/mL) for 24, 48, and 72 hours. Next, the medium of all wells was removed carefully, and 20 μL of MTT (1 mg/mL dissolved in PBS) and 180 µL of DMEM was added to each well and kept for 4.5 hours in the dark for further incubation. After removing content of the wells, the cells were washed twice with Phosphate Buffer Saline (PBS) and 200 μL of pure DMSO was added to the wells, and the absorbance of each well was read at 490 nm using a microplate reader (BioTek® ELx800, USA) during 15 to 30 minutes. For data analysis, mean Optical Density (OD) of each well was determined, and percentage of cell viability was calculated using the following formula: 

Cytotoxicity %=1-Mean absorbance of sampleMean absorbance of control×100 Viability%=100-cytotoxicity%

Lastly, using the Excel software a graph was plotted and the 50% inhibition concentration (IC50) values of silibinin on MCF-7 cells was determined on the graph. After determination of IC50, for evaluating the effect of silibinin on maspin and ERα expression, 3×10^5^ cells/wells were treated in a six-well plate with different sub-toxic concentrations of silibinin (50, 100, 150, 200, and 250 μg/mL) for 24 hours. Furthermore, cells with culture medium containing 10% DMSO were considered as a vehicle control. Finally, cells were incubated at 37°C in %5 CO_2_ for 24 hours.

**RNA Extraction and cDNA Synthesis**

Total RNA was extracted using a high pure RNA isolation kit (RNeasy® Plus Mini Kit, Quiagen, Maryland, USA), which includes a gDNA eliminator spin to remove DNA traces, according to the manufacturer’s instructions. The quantity and purity of the extracted RNA were measured using a NanoDrop spectrophotometer (Termoscientific, USA) at OD_260_ and OD_280_, and its integrity was checked on 1% agarose gel electrophoresis. First-strand of complementary DNA (cDNA) was generated from 1 μg of total RNA using an oligo (dT) 18 primer with superscript II reverse transcriptase kit (Takara, Japan), according to the manufacturer’s 

recommendations. The generated cDNA was used immediately for real-time PCR or stored at –20°C for later use.

**Gene Expression Analysis by Real-Time Polymerase Chain Reaction**

Quantitative real-time Reverse Transcription-PCR (qRT-PCR) was performed with SYBR Premix Ex Taq^TM^ II (Tli RNaseH plus) on an ABI thermal cycler instrument (Applied Biosystem 7500 Fast Real-Time PCR System, USA), according to the manufacturers' protocol. The amplification reaction was performed in a 20-μL volume containing 10 μL of SYBR Green PCR Master Mix, 2 μL of cDNA (50 ng/µL), 0.8 μL of forward and reverse primers (10 μM), 6 μL of nuclease-free water (Qiagen, Hilden, Germany) and 0.4 µL ROX Reference dye II. Thermal cycling conditions were as follows: preliminary denaturation at 95°C for 30 seconds, followed by 40 cycles of denaturation at 95°C for 30 seconds and a combined annealing/extension at 60°C for 3 seconds. The PCR amplification was performed with β-actin, as an internal reference gene. Specific oligonucleotide primers were designed from coding regions of human maspin, ER-α and β-actin cDNA (Gene ID: 5268, 2099, and 60, respectively) are presented in Table 1.

**Figure 1:**
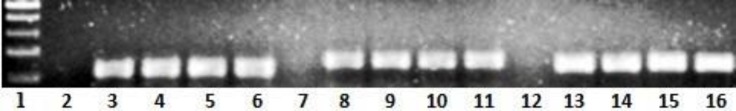
Representative Gel Picture of Real-Time Reverse Transcription-Polymerase Chain Reaction for Maspin, ERα, and β-actin in MCF-7 Cell Line

The specificity of amplified target genes was verified by agarose gel electrophoresis and melting curve analysis, as shown in Figure 1.

**Table 1:** Primers Used for Real-Time Polymerase Chain Reaction Amplifications

Primers	Primer length	Sequence (5^ʹ^3ʹ)	PCR Product size (bp)
ERα Forward	20	GCCCTACTACCTGGAGAAGG	117	
ERα Reverse	20	CTGGCCAATCTTTCTCTGCC		
Maspin Forward	20	CTTGCCTGTTCCTTTTCCAC	151	
Maspin Reverse	20	TGGAGAGAAGAGGACATTGC		
β-actin Forward	20	TGGACTTCGAGCAAGAGATG	137	
β-actin reverse	20	GAAGGAAGGCTGGAAGAGTG		

Lane 1 shows the 100-bp DNA ladder; Lane 2 shows ERα NTC, Lanes 3, 4, 5 and 6 show ERα samples (117 bp), Lane 7 shows maspin NTC, Lanes 8, 9, 10 and 11 show Maspin samples (151 bp); Lane 12 shows β-actin NTC, and Lanes 13,14,15 and 16 show β-actin samples (137 bp).

 Relative Expression Software Tool (REST, 2009 v2.0.13) was used for analysis by comparative Ct method (2^–ΔΔCT^). For this purpose, primarily, the mean threshold cycle (CT) values were calculated for reference and target genes (23). Threshold cycle (CT) value was defined as the cycle number at which the fluorescence generated within a reaction crosses the fluorescence threshold. The difference of CT values of the target and the reference gene was presented as ΔCT. Finally, ΔΔCT was calculated as the difference of ΔCT values between paired specimens. The 2^-ΔΔCT^, which represents the exponential value of ΔCT, is considered as fold change difference in expression of target genes between treated and controlled samples.

**Statistical Analysis**

Using the SPSS software and one-way analysis of variance, statistical analyses were performed and a P of < 0.05 was considered statistically significant. Data are expressed as mean ± Standard Error of the Mean (SEM). The 50% inhibition concentration (IC50) values of silibinin on MCF-7 cells at different time intervals were determined by analyzing dose-dependent inhibition, using the statistical software packages of Excel and GraphPad 6 prism.

## Results

**Inhibition Effect of Silibinin on MCF-7 Viability**

**Figure 2:**
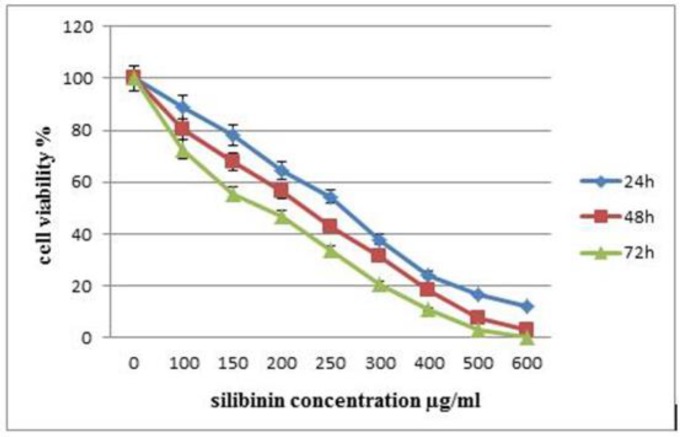
Effect of Silibinin on Metabolic Activity of MCF-7 Cell Line

The inhibitory effect of silibinin on metabolic activity of the MCF-7 cell line was investigated through the MTT assay at different concentrations and time intervals (24, 48 and 72 hours). The growth of the MCF-7 cell line was inhibited by silibinin in a dose- and time-dependent manner. As shown in Figure 2, compared to the controls, treatment of the MCF-7 cell line with silibinin at 100, 150, 200, 250, 300, 400, 500 and 600 µg/mL for 24 hours reduced the metabolic activity of MCF-7 cells by 11.25%, 21.89%, 35.51%, 45.57%, 62.14%, 75.74%, 83.84% and 88.17%, respectively.

The cells were treated with different concentrations of silibinin for 24, 48, and 72 hous and their metabolic activity was assessed by the MTT assay. Results are presented as percentage of metabolic activity compared to untreated control and are presented as mean ± SEM, from three independent experiments. 

Data analysis showed that IC50 of silibinin on MCF-7 breast cancer cell line was 296 μg/mL during the 24-hour time interval by the MTT assay. Incubation of the cells with silibinin for 48 and 72 hours significantly decreased IC50 values to 250 and 207 μg/mL (p = 0.005 and p= 0.006), respectively.

**Effect of Silibinin on ERα Gene Expression**

**Figure 3:**
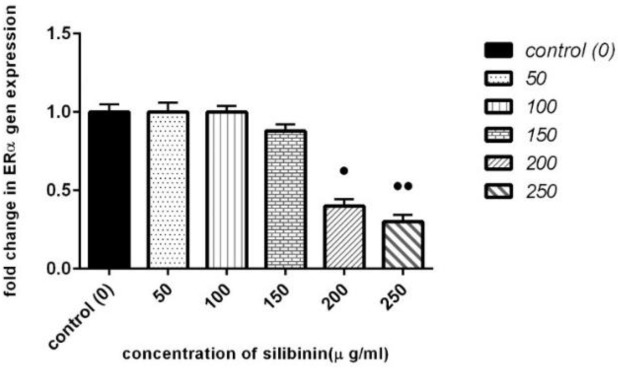
Effect of Silibinin on ERα Expression in MCF-7 cell line

The ERα gene expression in cancer cell line after exposure to silibinin at 50, 100, 150, 200 and 250 µg/mL after 24 hours of incubation was assessed by the real time PCR method. The results of the real-time PCR showed a signiﬁcant decrease in ERα gene expression in the treated cells compared to the controls (Figure 3). While no significant difference was detected for ERα expression in the presence of 50, 100, and 150 μg/mL of silibinin, at 24 hours, however, a signiﬁcant decrease was observed for ERα expression at 200 μg/mL (P = 0.036) and 250 μg/mL (p =0.008), respectively (Figure 3).

Increased silibinin concentration resulted in a signiﬁcant decrease of ERα expression. Values are given as mean ± SEM from three independent experiments. P values of < 0.05 are considered significant. 

**Effect of Silibinin on Maspin Gene Expression**

**Figure 4:**
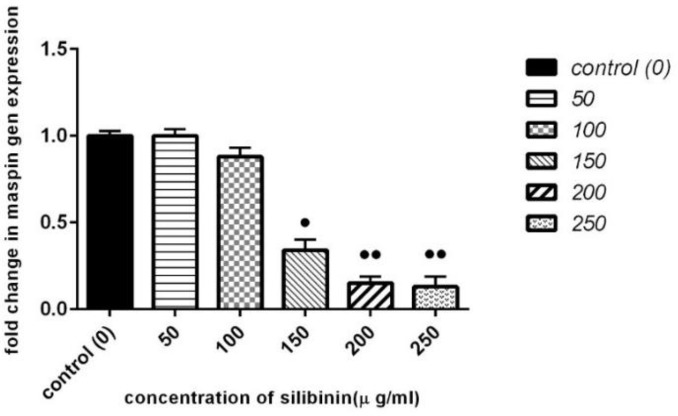
Effect of Silibinin on Maspin Expression in MCF-7 cell line

The expression of maspin mRNAs was determined using the real-time PCR method. The results of the real time PCR showed that expression of maspin in MCF-7 cell line was decreased by incubation at 50 to 250 µg/mL concentration of silibinin in a dose-dependent manner (Figure 4). 

Increased silibinin concentration resulted in a signiﬁcant decrease of maspin expression. Values are given as mean ± SEM from three independent experiments. P values of < 0.05 were considered significant.

Silibinin treatment (24 hours) reduced fold changes by 0.34 at a concentration of 150 µg/mL (P =0.008). The incubation of the cells with silibinin at 200 and 250 µg/mL decreased fold changes to 0.15 (p =0.005) and 0.13 (p =0.003), respectively. 

## Discussion

There is an increasing body of evidence that inactivation of metastatic suppressor genes plays a crucial role in breast cancer progression (24-26). In addition, in vitro and in vivo studies reported that maspin was a proposing candidate for treatment in breast cancer. Regarding the results, maspin expression may be induced by ER activity, which itself may be induced by tamoxifen (13). Among the conditions, in which chemotherapeutic drugs have shown a wide variety of side effects, plant-based drugs represent high chemo-preventive properties and low toxicity for normal tissue. One of these natural agents is silibinin, which has anti-metastatic and anti-cancer effects, in several types of cancers, including prostate and breast cancer. Nejati-Koshki et al. found that the level of leptin gene expression and secretion was decreased by silibinin in T47D cell line (27). In another study, Yousefi et al*. *reported that silibinin has a high inhibitory effect on cell proliferation and activation of NF-κB in ER-negative breast carcinoma cells (28). Tyagi et al*.* reported that silibinin together with doxorubicin have the strongest inhibitory effects on cell growth compared to each agent alone in MCF-7 cell line (29). In the current study, it was found that silibinin decreases cell viability, maspin and ERα gene expression in a dose- and time-dependent manner in MCF-7 cell line.

The silibinin concentrations used in the current study for the assessment of cell viability were 100, 150, 200, 250, 300, 400, 500, and 600 µg/mL, respectively, which were selected based on a previous study (30). No effect on cell viability was observed at low concentrations (<100 µg/mL). However, at high concentrations (>300 µg/mL), cell viability significantly decreased to approximately 62% compared to the controls. These results showed a potent reduction in MCF-7 cell viability in a time- and dose- dependent manner, which is in agreement with the results of a previous study (30). In our study, the IC50 value of silibinin for this cell line during a 24-hour time interval was determined as 296 μg/mL, therefore, the concentrations of silibinin under IC50 were used for the assessment of gene expression. 

Real time PCR was used to evaluate fold changes of maspin and ERα expression at mRNA level in ER-positive MCF-7 cells. While, no significant difference was observed for ERα mRNA level between treated and controlled cells at the 150 µg/mL concentration of silibinin, however, a signiﬁcant decrease was observed for maspin at 150 µg/mL. Our findings showed that exposure to silibinin at 200 µg/mL and 250 µg/mL resulted in a reduction of ERα expression, which was accompanied by a reduction of maspin expression. According to the results, silibinin-induced reduction of maspin expression in MCF-7 breast cancer cell line seemed to be accompanied by reduction of ERα expression. This result is important, because it has been seen in the majority of breast carcinomas ERα is highly expressed than normal tissues (31). In addition, the proliferation-stimulatory effect of estrogens is mediated through the activation of ERα in ERα-positive breast cancer (32). Therefore, targeting the ERα in breast cancers could be a promising step for its treatment. Maspin is often known as a tumor suppressor gene and inhibits cell motility, invasion, and metastasis in human breast carcinoma (24-26). Previous studies have shown that maspin expression was correlated with large tumor size in breast cancer and with a high tumor grade (6, 7). Conversely, we observed that maspin expression in MCF-7 cell line was decreased after treatment with silibinin (Figure 4). Therefore, further investigations are needed to confirm the tumor suppressor function of maspin in breast cancer.

Previous studies indicated that the effect of tamoxifen on the activation of tumor suppressor maspin gene in breast cancer is mediated through ERα (13, 33). Our findings demonstrated that silibinin could inhibit ERα gene expression in MCF-7 breast cancer cell line, which is consistent with the results reported by Zheng et al. (34). They found that treatment of ERa-positive MCF-7 cell line with silibinin down-regulates the expression of ERα, which plays a key role in silibinin-induced apoptosis in these cells (34). Biochemical and cellular evidence showed that P53 directly regulates the expression of maspin; unfortunately, the expression of p53 has not been measured in our study. Chandra et al. analyzed the expression of maspin protein in the MCF-7 and MDA-MB-231 cell lines treated with curcumin, and observed that curcumin induced the expression of maspin gene at both mRNA and protein levels in MCF-7 cell line (wild- type p53), in a time-dependent manner. However, these results were not observed in MDA-MB-231 cell line (mutant p53) (11). In addition, previous studies have demonstrated that p53 activates the transcription of ERα promoter in human breast cancer (35). Pirouzpanah et al. reported that silibinin increases p53 gene expression and induces cell cycle arrest and apoptosis in MCF-7 cell line (36). These studies showed that p53 is necessary for the reduction of maspin and ERα gene expression in MCF-7 breast cancer cell line by silibinin. Further studies are needed to explore molecular mechanism(s) involved in silibinin-induced reduction of maspin and ERα gene expression in the breast cancer cell line. 

## Conclusion

The results indicated that treatment of MCF-7 human breast cancer cell line with silibinin decreases cell viability, maspin, and ERα gene expression in a dose- and time-dependent manner. To the best of our knowledge this is the first report that addresses the effect of silibinin on maspin expression in MCF-7 breast cancer cell line. The therapeutic effect of silibinin on the treatment of breast cancer may be mediated by reduction of ERα expression, and further studies in this direction are necessary for identifying therapeutic implications of silibinin.

## References

[1] Zou Z, Anisowicz A, Hendrix MJ, Thor A, Neveu M, Sheng S (1994). Maspin, a serpin with tumor-suppressing activity in human mammary epithelial cells. Science.

[2] Benarafa C, Remold-O'Donnell E (2005). The ovalbumin serpins revisited: perspective from the chicken genome of clade B serpin evolution in vertebrates. Proc Natl Acad Sci U S A.

[3] Latha K, Zhang W, Cella N, Shi HY, Zhang M (2005). Maspin mediates increased tumor cell apoptosis upon induction of the mitochondrial permeability transition. Mol Cell Biol.

[4] Li Z, Shi HY, Zhang M (2005). Targeted expression of maspin in tumor vasculatures induces endothelial cell apoptosis. Oncogene.

[5] Schaefer JS, Zhang M (2003). Role of maspin in tumor metastasis and angiogenesis. Curr Mol Med.

[6] Kim DH, Yoon DS, Dooley WC, Nam ES, Ryu JW, Jung KC (2003). Association of maspin expression with the high histological grade and lymphocyte-rich stroma in early-stage breast cancer. Histopathology.

[7] Umekita Y, Yoshida H (2003). Expression of maspin is up-regulated during the progression of mammary ductal carcinoma. Histopathology.

[8] Maass N, Hojo T, Rosel F, Ikeda T, Jonat W, Nagasaki K (2001). Down regulation of the tumor suppressor gene maspin in breast carcinoma is associated with a higher risk of distant metastasis. Clin biochem.

[9] Odero-Marah VA, Khalkhali-Ellis Z, Chunthapong J, Amir S, Seftor RE, Seftor EA (2003). Maspin regulates different signaling pathways for motility and adhesion in aggressive breast cancer cells. Cancer Biol Ther.

[10] Zhang M, Volpert O, Shi YH, Bouck N (2000). Maspin is an angiogenesis inhibitor. Nature medicine.

[11] Prasad CP, Rath G, Mathur S, Bhatnagar D, Ralhan R (2010). Expression analysis of maspin in invasive ductal carcinoma of breast and modulation of its expression by curcumin in breast cancer cell lines. Chem Biol Interact.

[12] Bièche I, Onody P, Tozlu S, Driouch K, Vidaud M, Lidereau R (2003). Prognostic value of ERBB family mRNA expression in breast carcinomas. Int J Cancer.

[13] Liu Z, Shi HY, Nawaz Z, Zhang M (2004). Tamoxifen induces the expression of maspin through estrogen receptor-alpha. Cancer lett.

[14] Zhang M, Magit D, Sager R (1997). Expression of maspin in prostate cells is regulated by a positive ets element and a negative hormonal responsive element site recognized by androgen receptor. Proc Natl Acad Sci U S A.

[15] Momeny M, Khorramizadeh MR, Ghaffari SH, Yousefi M, Yekaninejad MS, Esmaeili R (2008). Effects of silibinin on cell growth and invasive properties of a human hepatocellular carcinoma cell line, HepG-2, through inhibition of extracellular signal-regulated kinase 1/2 phosphorylation. Eur J Pharmacol.

[16] Ting H, Deep G, Agarwal R (2013). Molecular mechanisms of silibinin-mediated cancer chemoprevention with major emphasis on prostate cancer. AAPS J.

[17] Cheung CW, Taylor PJ, Kirkpatrick CM, Vesey DA, Gobe GC, Winterford C (2007). Therapeutic value of orally administered silibinin in renal cell carcinoma: manipulation of insulin-like growth factor binding protein-3 levels. BJU Int.

[18] Yang SH, Lin JK, Chen WS, Chiu JH (2003). Anti-angiogenic effect of silymarin on colon cancer LoVo cell line. J Surg Res.

[19] Chu SC, Chiou HL, Chen PN, Yang SF, Hsieh YS (2004). Silibinin inhibits the invasion of human lung cancer cells via decreased productions of urokinase-plasminogen activator and matrix metalloproteinase-2. Mol Carcinog.

[20] Katiyar SK, Korman NJ, Mukhtar H, Agarwal R (1997). Protective effects of silymarin against photocarcinogenesis in a mouse skin model. J Natl Cancer Inst.

[21] Flaig TW, Gustafson DL, Su LJ, Zirrolli JA, Crighton F, Harrison GS (2007). A phase I and pharmacokinetic study of silybin-phytosome in prostate cancer patients. Invest New Drugs.

[22] Deep G, Agarwal R (2010). Antimetastatic efficacy of silibinin: molecular mechanisms and therapeutic potential against cancer. Cancer Metastasis Rev.

[23] Livak KJ, Schmittgen TD (2001). Analysis of relative gene expression data using real-time quantitative PCR and the 2(-Delta Delta C(T)) Method. Methods.

[24] Noh EM, Yi MS, Youn HJ, Lee BK, Lee YR, Han JH (2011). Silibinin enhances ultraviolet B-induced apoptosis in mcf-7 human breast cancer cells. J Breast Cancer.

[25] Moerkens M, Zhang Y, Wester L, van de Water B, Meerman JH (2014). Epidermal growth factor receptor signalling in human breast cancer cells operates parallel to estrogen receptor alpha signalling and results in tamoxifen insensitive proliferation. BMC cancer.

[26] Williams C, Lin CY (2013). Oestrogen receptors in breast cancer: basic mechanisms and clinical implications. Ecancermedicalscience.

[27] Nejati-Koshki K, Zarghami N, Pourhassan-Moghaddam M, Rahmati-Yamchi M, Mollazade M, Nasiri M (2012). Inhibition of leptin gene expression and secretion by silibinin: possible role of estrogen receptors. Cytotechnology.

[28] Yousefi M, Ghaffari SH, Zekri A, Hassani S, Alimoghaddam K, Ghavamzadeh A (2014). Silibinin induces apoptosis and inhibits proliferation of estrogen receptor (ER)-negative breast carcinoma cells through suppression of nuclear factor kappa B activation. Arch Iran Med.

[29] Tyagi AK, Agarwal C, Chan DC, Agarwal R (2004). Synergistic anti-cancer effects of silibinin with conventional cytotoxic agents doxorubicin, cisplatin and carboplatin against human breast carcinoma MCF-7 and MDA-MB468 cells. Oncol Rep.

[30] Stark AM, Schem C, Maass N, Hugo HH, Jonat W, Mehdorn HM (2010). Expression of metastasis suppressor gene maspin is reduced in breast cancer brain metastases and correlates with the estrogen receptor status. Neurol Res.

[31] Mohsin SK, Zhang M, Clark GM, Craig Allred D (2003). Maspin expression in invasive breast cancer: association with other prognostic factors. J Pathol.

[32] Stark AM, Tongers K, Maass N, Mehdorn HM, Held-Feindt J (2005). Reduced metastasis-suppressor gene mRNA-expression in breast cancer brain metastases. J Cancer Res Clin Oncol.

[33] Khalkhali-Ellis Z, Christian AL, Kirschmann DA, Edwards EM, Rezaie-Thompson M, Vasef MA (2004). Regulating the tumor suppressor gene maspin in breast cancer cells: a potential mechanism for the anticancer properties of tamoxifen. Clin Cancer Res.

[34] Zheng N, Zhang P, Huang H, Liu W, Hayashi T, Zang L (2015). ERa down-regulation plays a key role in silibinin-induced autophagy and apoptosis in human breast cancer MCF-7 cells. J Pharmacol Sci.

[35] Shirley SH, Rundhaug JE, Tian J, Cullinan-Ammann N, Lambertz I, Conti CJ (2009). Transcriptional regulation of estrogen receptor-alpha by p53 in human breast cancer cells. Cancer Res.

[36] Pirouzpanah MB, Sabzichi M, Pirouzpanah S, Samadi N (2015). Silibilin-Induces Apoptosis in Breast Cancer Cells by Modulating p53, p21, Bak and Bcl-xl Pathways. Asian Pac J Cancer Prev.

